# Ewing Sarcoma Protein: A Key Player in Human Cancer

**DOI:** 10.1155/2013/642853

**Published:** 2013-09-03

**Authors:** Maria Paola Paronetto

**Affiliations:** ^1^Department of Health Sciences, University of Rome “Foro Italico”, 00135 Rome, Italy; ^2^Laboratory of Cellular and Molecular Neurobiology, Fondazione Santa Lucia IRCSS, 00143 Rome, Italy

## Abstract

The Ewing sarcoma protein (EWS) is a well-known player in cancer biology for the specific translocations occurring in sarcomas. The EWS-FLI1 gene fusion is the prototypical translocation that encodes the aberrant, chimeric transcription factor, which is a landmark of Ewing tumors. In all described Ewing sarcoma oncogenes, the EWS RNA binding domains are completely missing; thus RNA binding properties are not retained in the hybrid proteins. However, it is currently unknown whether the absence of EWS function in RNA metabolism plays a role in oncogenic transformation or if EWS plays a role by itself in cancer development besides its contribution to the translocation. In this regard, recent reports have highlighted an essential role for EWS in the regulation of DNA damage response (DDR), a process that counteracts genome stability and is often deregulated in cancer cells. The first part of this review will describe the structural features of EWS and its multiple roles in the regulation of gene expression, which are exerted by coordinating different steps in the synthesis and processing of pre-mRNAs. The second part will examine the role of EWS in the regulation of DDR- and cancer-related genes, with potential implications in cancer therapies. Finally, recent advances on the involvement of EWS in neuromuscular disorders will be discussed. Collectively, the information reviewed herein highlights the broad role of EWS in bridging different cellular processes and underlines the contribution of EWS to genome stability and proper cell-cycle progression in higher eukaryotic cells.

## 1. Introduction

EWS was originally identified because of the t(ll;22) (q24;q12) chromosome translocation, characteristic of Ewing sarcoma and related subtypes of primitive neuroectodermal tumors [1]. In these types of cancers, a hybrid transcript is generated by the genomic regions where the breakpoints of chromosome 22 and 11 occur. The translocation alters the open reading frame of the *EWSR1 *gene on chromosome 22 by substituting a sequence encoding a putative RNA-binding domain with that of the DNA-binding domain of *FLI-1*, the human homologue of murine *Fli-1* [2]. Another translocation involving the *EWSR1* gene was subsequently identified in malignant melanoma of soft parts: the deduced chimaeric protein consisted of the N-terminal domain of EWS linked to the bZIP domain of ATF-1, a transcription factor regulated by cAMP that was not previously implicated in oncogenesis [3]. In addition to the EWS-FLI-1 and EWS-ATF-1 fusions, the *EWSR1* gene can be fused to *ERG*, which encodes a transcription factor closely related to FLI-1 but located on chromosome 21 [4]. Thus, the oncogenic conversion of EWS follows a common scheme of activation, by exchanging its RNA binding domain with different DNA binding domains, thus generating tumor-specific fusions proteins. The oncogenes generated in Ewing sarcoma have been extensively studied and the main aspects of Ewing sarcoma have been covered by several excellent recent reviews [5–8]. On the contrary, only limited information is available on the function of the EWS protein itself and whether or not it plays a role in oncogenesis or in other pathological situations. 

This review will focus on the role played by EWS protein in the regulation of RNA metabolism, in normal conditions as well as in pathological situations.

## 2. EWS Family

**Figure 1:**
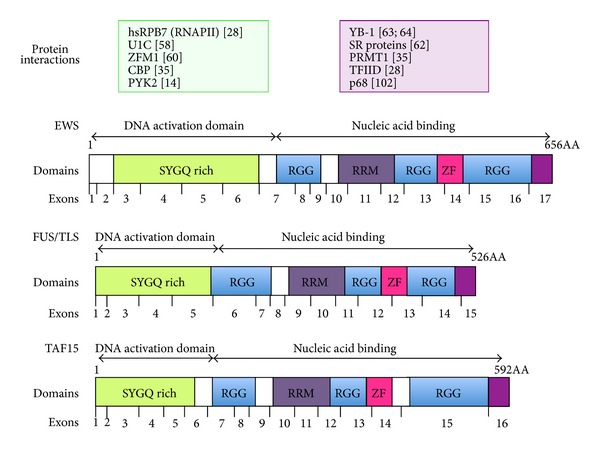
Domain structure of TET proteins: the N-terminal activation domain and the C-terminal nucleic acid binding domain of TET proteins are schematized: the SYGQ rich domain, RGG boxes, and the RNA binding domain (RBD), Cys2-Cys2 zinc finger (ZF). Below the scheme, the exons encoding each domain are indicated for EWS, FUS/TLS, and TAF15, respectively [17, 18]. In the upper part of the figure the proteins that have been described to interact with either the DNA activation domain or the nucleic acid binding domain of EWS are listed.

EWS belongs to the TET family of DNA and RNA-binding proteins, which consists of translocated in liposarcoma/fused in sarcoma protein (FUS/TLS), Ewing sarcoma protein (EWS), and TATA-binding protein-associated factor 15 (TAF15). TET proteins bind RNA as well as DNA and are implicated in the regulation of gene expression and in cell signaling. In addition to mRNA splicing and processing, TET proteins also directly interact with transcription factors and can function as transcriptional cofactors. Moreover, like EWS, all the members of the TET family also contribute to human pathologies, as they are involved in sarcoma translocations [2, 8] and neurological diseases [9, 10]. They contain several conserved domains: a serine-tyrosine-glycine-glutamine (SYGQ) domain, an RNA-recognition motif (RRM), a zinc finger motif, and three RNA binding Arginine-Glycine-Glycine- (RGG-) rich domains (Figure 1) [11]. The TET proteins are ubiquitously expressed in most human tissues; they have a nuclear localization and predominantly reside in the nucleus [12, 13] but can translocate to the cytoplasm upon stress conditions [13, 14] or in pathological situations [15, 16]. 

## 3. Genomic Structure of *EWSR1* Gene

The *EWSR1* gene spans about 40 kb on chromosome 22 and is encoded by 17 exons [17]. The first 7 exons encode the N-terminal domain of EWS, which consists of a repeated degenerated polypeptide of 7 to 12 residues rich in tyrosine, serine, threonine, glycine, and glutamine (SYGQ). The degenerated SYGQ repeat [19] is encoded by sequences that stretch from exon 3 to the end of exon 7. Exons 8 to 17 of EWS encode regions associated with RNA binding. The three RGG motifs are mainly encoded by exons 8, 9, 14, and 16, while exons 11, 12, and 13 encode the RRM (Figure 1). The DNA sequence in the 5′ region of the *EWSR1* gene lacks canonical promoter elements, such as TATA and CCAAT consensus sequences [17], but contains G/C rich stretches [18], suggesting a housekeeping role for TET proteins.

*TAF15, FUS/TLS,* and *EWSR1* genes display extensive similarities, suggesting that they are closely related and may have originated from a common ancestor gene (Figure 1) [18]. Although their total genomic size differs, TET genes share a similar genomic organization with exon/intron junctions at the same sites, reflecting the conserved protein structure consisting of an N-terminal transcription activation domain and the RRM, zinc finger domain, and several RGG repeat boxes at the C-terminus (Figure 1). The first exon of these three genes encodes a 5′ untranslated region and the translation initiation codon. The RRM region and flanking parts show extensive homologies in amino acid composition and exon/intron structure. On the other hand, the parts of the genes located between *FUS/TLS* exons 4, 5, and 6, *TAF15* exons 5, 6 and 7, and *EWSR1* exons 5, 6, 7, and 8 display lower degree of homology.

Different isoforms of the EWS protein have been described. A splicing isoform containing the exon 4A is specifically expressed in the central nervous system (isoN), in both mice and humans, and is not present in the tumor-specific EWS fusions [20]. Interestingly, the regions flanking this variable exon contain many putative PTB binding sites [20], highlighting the possibility that EWS alternative splicing (AS) could be target of PTB/nPTB regulation in neurons. Notably, whereas the level of isoN expression is stable from embryonic stage E11.5 to birth, the level of the main isoform shows a considerable decrease. This suggests that the two isoforms play distinct roles during brain development and that the isoN variant is associated with neural differentiation [20]. Additional isoforms lacking exon 6 and/or including exon 8A have been sequenced from a pool of 107 human cDNAs and annotated [21]. Notably, this triplet encodes Serine 325 [22], which is a putative casein kinase II phosphorylation site and may represent a posttranslational modification that distinguishes between the two protein isoforms of EWS.

## 4. Physiological Functions of the EWS Protein

The functions of the EWS protein have been partially revealed by the generation of a knockout mouse model through a gene targeting strategy [23].* Ews* ^−/−^ mice were born at the expected mendelian ratio, but displayed smaller size than their littermates and have a high rate (about 90%) of postnatal mortality prior to weaning. Analysis of these mice also demonstrated that the *Ewsr1* gene is essential for pre-B lymphocyte development and meiosis. Interestingly, *Fus/Tls*-null mice showed a quite similar phenotype [24, 25]; however, while FUS/TLS is required for pairing of the autosomes, EWS appears to be involved in pairing the XY sex chromosomes during meiosis [23]. Indeed, *Ews*-null spermatocytes show a high frequency of unsynapsed XY chromosomes but display normal formation of autosomal bivalents; in contrast, defects in the formation of autosomal bivalents were observed in* Fus/Tls*-deficient spermatocytes. Moreover, while EWS expression is diffuse throughout spermatocytes, FUS/TLS expression is excluded from the sex body region [23, 25]. Nevertheless, as a result of these defects in chromosome pairing, both knockout mice are sterile.

Interestingly, inactivation of *Ews* in mouse embryonic fibroblasts resulted in hypersensitivity to ionizing radiation and caused premature cellular senescence, possibly due to lack of the reported interaction of the EWS protein with nuclear lamin A/C [23]. Moreover, loss of *Ews* brought about dramatic changes in the dynamics of hematopoietic stem cells (HSCs). In particular, deletion of *Ews* promoted stem cell exit from a quiescent state and entrance into the cell cycle, which is one of the common features found in HSCs from aged wild-type mice. Cellular senescence is associated with a progressive impairment of the immune system or “immunosenescence,” which is characterized by a defective Th1 (T helper 1) response. T cells can be separated depending upon the specific cytokines they secrete in response to antigenic stimulation. Th1 cells primarily produce interferon- (IFN-) *γ* and interleukin- (IL-) 2 [26]. In line with this notion, *Ews* ^−/−^ mice displayed a specific decrease in IFN-*γ* and IL-2 production [27]. Thus, the phenotype of *Ews* ablation mouse models is consistent with a physiological function for EWS in response to the occurrence of genomic alterations, as suggested by the hypersensitivity to IR in the absence of EWS and the defects in cell types where physiological DNA breaks occur, including B cells engaged in VDJ recombination and meiotic germ cells engaged in homologous recombination. Collectively these results highlight a role for EWS in the response to DNA damage.

## 5. EWS and Transcription

Several observations document the involvement of EWS in transcriptional regulation of gene expression. EWS is able to associate with different subunits of the transcription factor TFIID [28]. Notably, this feature is not maintained by the oncogenic fusion protein EWS-FLI-1, suggesting that EWS and EWS-FLI-1 behave differently in this respect. Moreover, EWS associates directly with RNA Polymerase II (RNAP II) [28, 29] and with its subunits Rpb3, Rpb4, and Rpb7 [28, 30]. In addition to directly contacting general transcription factors and RNAPII, EWS protein regulates transcription through the interaction with activators and repressors. For instance, EWS binds various transcription factors containing the POU (from the mammalian Pit-1 and Oct-1, and the *C. elegans* Unc86) homeodomain, a specific DNA-binding domain conserved throughout evolution that appears to exert critical developmental functions. EWS binds OCT4, a transcriptional activator that is essential to maintain an undifferentiated totipotent state of embryonic stem and germ cells [31], and Brn3a, the brain-specific homeobox/POU domain protein 3A, which is expressed in the developing and adult nervous systems and promotes neuronal differentiation [32, 33]. Additionally, EWS has been shown to interact with the histone acetyl-transferase CREB-binding protein (CBP) and the p300 transcriptional activator, thus cotransactivating *in vitro* several promoters in a cell-type specific manner [34, 35]. Collectively these studies indicate that EWS may regulate transcription both positively and negatively.

The amino terminus of EWS protein was shown to act as a transcriptional activator when fused to a DNA-binding domain [36]. However, the transcription activation potential is decreased or even abolished in the full-length protein, indicating that the RNA or DNA binding domains may regulate the activation domain [37, 38]. Deletions of either the zinc finger domain or the RRM play no role in repression. In contrast, deletion of each RGG domain results in substantial loss of repression, indicating that the RGG regions are necessary for repression [37, 38]. Thus, the transcription repression might result from the C-terminal RGG domain that blocks the interaction between the glutamine-rich domain in the N-terminus and hsRPB7 (human homologue of the seventh largest subunit of yeast RNAPII (RPB7) (Figure 1, upper panel).

## 6. EWS and Nucleic Acid Binding

As mentioned above, EWS protein contains several domains capable to bind independently nucleic acid sequences. The RRM, a classical RNA Binding Domain (RBD), is the most conserved region within the TET protein family. The RRM is composed of approximately 90 amino acids and contains a central sequence of eight conserved residues that are mainly aromatic and positively charged. The RRM folds into a sandwich structure composed of one four-stranded antiparallel *β*-sheet and two *α*-helices (*α*1 and *α*2) packed against the *β*-sheet [39]. Within this domain, two short ribonucleoprotein motifs, RNP-1 (in *β*3-strand) and RNP-2 (in *β*1-strand) usually separated by 25–35 residues, directly contact RNA via hydrogen bonds and ring stacking [39]. Unlike other proteins with this type of RBD, TET proteins have an acidic residue at the second position and a threonine residue in the fourth position of RNP-1, as well as an unusually long loop after the first *α*-helix [40], which may affect the structure of the protein since this region contributes to the hydrophobic core of the domain and to RNA-binding specificity or affinity. 

The (DW) C4 zinc finger motif has several highly conserved features, distinct from those present in other known C5-C5 zinc-fingers [41]. It contains an aspartic acid (D) followed by tryptophan (W), preceding the first cysteine (C) residue of the finger. Within the zinc-finger loop, the fourth residue after the second cysteine is an asparagine and is followed by an aromatic residue. Secondary structure prediction algorithms suggest that the C-terminal region of the motif may give rise to an alpha helix, a general feature of many zinc fingers motif [42]. 

The carboxy-terminus of EWS contains also three RGG motifs that may increase RNA affinity of the RRM or zinc finger and may also be the site of posttranslational modifications that regulate RNA binding or protein-protein interactions [39]. Sequence-specific RNA binding by EWS has been examined by various groups. It was shown that EWS binds polyU and polyG sequences through its carboxy-terminal RGG domain [43]. Moreover, *in vitro* systematic evolution of ligands by exponential enrichment (SELEX) [44] experiments suggested that the RRM and RGG motifs of TET proteins cooperate to bind GGUG RNA, although with relatively low affinity (*Kd* = 250 nM) [45].

RGG domains are present in several RNA and DNA binding proteins. The RGG domain of FMRP, an RNA binding protein involved in neural cell differentiation, interacts with RNA forming G-quartets, a unique structural arrangement intrinsic to guanine-rich DNA and RNA [46]. In addition, the RRM and the RGG domains of nucleolin, a DNA-binding protein contributing to the transcription of ribosomal RNA, bind to ribosomal DNA forming G-quartets [47]. EWS protein specifically targets DNA and RNA forming G-quadruplex* in vitro*, the four-stranded nucleic acid structures folded from G-rich repeat sequences stabilized by the stacking of planar G-quartets. The specificity of G-quadruplex recognition depends on the guanidinium group of the arginine in the RGG domain of the C-terminus of EWS [48]. 

It has been reported that overexpression of EWS leads to the activation of the *c-fos*, *Xvent-2,* and *ErbB2* promoters [12], but whether the role of EWS in transcription is determined by its ability to target a specific DNA and RNA structure or only by binding to the RNAPII is not clear. Notably, the *c-fos* and *ErbB2* promoters contain G-rich sequences that could potentially form G-quadruplex structures [49].

Nevertheless, DNA binding is likely to occur also through the zinc finger, a domain frequently found in transcription factors [50]. Indeed, mobility shift experiments revealed that EWS binds to single-stranded (but not to double-stranded) DNA, with preference for its target exon sequences [51], opening the possibility that EWS is recruited during transcription through specific recognition of DNA and/or RNA sequences.

Lastly, posttranslational modifications can affect binding of EWS to nucleic acids. It has been shown that EWS IQ domain, a 25-amino-acid domain located at the end of the activation domain, interacts with calmodulin and is phosphorylated by protein kinase C [52]. The IQ domain forms an amphiphilic seven-turn *α*-helix capable of binding calmodulin in a Ca^2+^-independent manner, a well-characterized feature of neuronal proteins such as neuromodulin and neurogranin [52]. Interestingly, PKC phosphorylation of EWS within the IQ domain inhibits its binding to RNA homopolymers, and, conversely, RNA binding to EWS interferes with PKC phosphorylation [53]. Moreover, enzymatic methylation of arginine residues by protein arginine N-methyltransferase 3 (PRMT3) reduces the affinity of EWS RGG (RGG3) domain toward G-quadruplex, while it increases its binding to single-strand DNA and RNA. Conversely, replacement of the arginine methylated by PRMT3 with lysine residues within the RGG domains decreases the binding affinity of EWS toward G-quadruplex DNA and RNA [53]. Furthermore, EWS methylation by PRMT1 (a homologue of PRMT3) reduces CBP-dependent EWS transcriptional activity and induces EWS translocation into the cytoplasm [54]. Thus, these observations suggest that several posttranslational modifications can fine-tune the affinity of EWS for its nucleic acid targets.

## 7. EWS and Splicing

Many reports have highlighted a potential role for EWS in splicing regulation. Most human genes contain introns that are removed by splicing, a process orchestrated and catalyzed by a large multiprotein/RNA complex named spliceosome. The spliceosome is composed of five small nuclear RNAs (snRNAs)—U1, U2, U4, U5, and U6 snRNA—as well as many protein factors [55]. Some of these proteins are tightly associated with the snRNAs, forming small nuclear ribonucleoproteins (snRNPs) assembled in a stepwise manner onto the pre-mRNA [55]. Work over the last two decades has elucidated the temporal sequence of recognition of the splice sites by the respective snRNPs and protein factors and the identity of former proteins of the spliceosome. EWS and FUS/TLS were identified by enhanced mass spectrometric tools and improved databases as proteins copurifying with splicing complexes assembled on pre-mRNAs [56]. Moreover, *in vitro* snRNP reconstitution and snRNP immunoprecipitation experiments have demonstrated, at least for FUS/TLS, the association with the Sm core domain of the various spliceosomal snRNPs [57].

Regarding EWS, a yeast two-hybrid screen revealed that it interacts with U1C, one of the three U1 small nuclear protein component of the U1snRNP [58], which binds to the 5′ splice site on pre-mRNA to form a stable complex identified as the early (E) complex in mammalian splicing extracts [59]. On the other hand, EWS has also been shown to interact with ZFM1, a transcriptional repressor identical to the branch-point binding protein BBP/SF1 [60], but whether or not it is involved in the recognition of 3′ splice site, as demonstrated for FUS/TLS during the second step of splicing [61], is still unknown. 

As described above, EWS protein binds to hyperphosphorylated RNAPII through the N-terminal domain and recruits serine-arginine (SR) splicing factors through the C-terminal domain, thus coupling transcription to RNA splicing [62] (Figure 1, upper panel). The C-terminal domain is also involved in the interaction with YB-1, a shuttling splicing activator also playing a role in mRNA stability and translation [63, 64]. It has been shown that the recruitment of processing factors and the maturation of pre-mRNAs occur, at least in part, cotranscriptionally and is enhanced by RNAPII and by its phosphorylation [65]. On one hand, transcriptional coregulators are recruited by transcription factors to their target genes thus affecting splicing decisions; on the other hand, transcriptional coregulators can modulate the rate of transcription elongation, which in turn affects AS decisions [65]. Thus, recruitment of splicing factors through interactions with the transcription machinery, as for EWS, is one mechanism by which coupling between transcription and AS occurs.

Consistent with this potential dual role, EWS has been shown to regulate cyclin D1 transcripts both transcriptionally and at the level of splicing [66]. Human cyclin D1 (*CCND1* gene) is expressed as two isoforms derived by AS, termed D1a and D1b, which differ for the inclusion of intron 4 in the D1b mRNA [67]. Both isoforms are frequently upregulated in human cancers, but cyclin D1b displays relatively higher oncogenic potential [68]. It has been shown that EWS modulates AS by altering RNAPII dynamics (phosphorylation and speed) over the *CCND1* gene [69]. EWS increases the speed of elongating RNAPII over the *CCND1* gene, and EWS depletion decreases RNAPII occupancy, but not Serine-5 phosphorylation at the 5′ end of the *CCND1* gene, thus decreasing cyclin D1a but not D1b mRNA levels [66]. 

**Figure 2:**
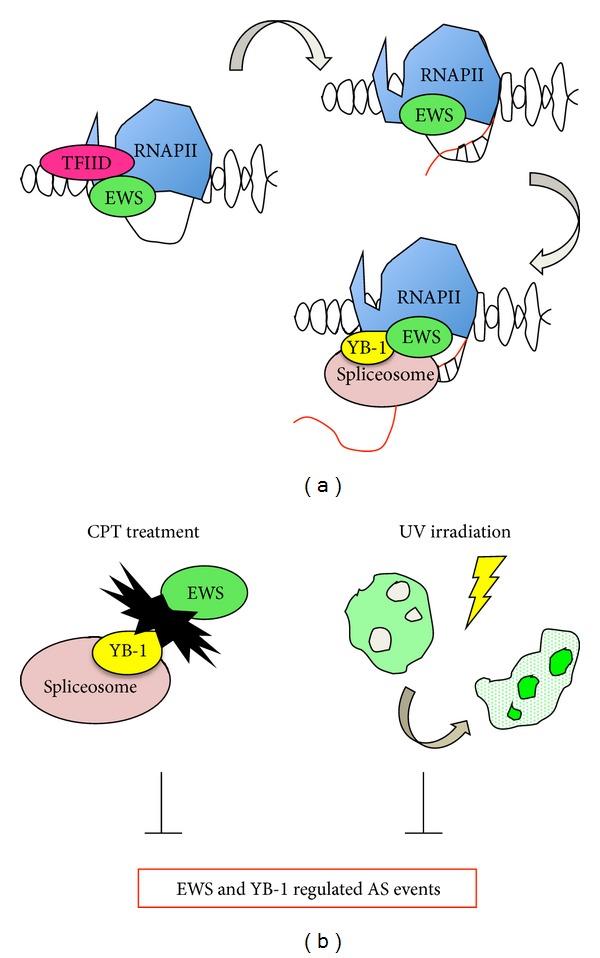
Schematic representation of the cross-talk between the transcriptional and splicing machineries mediated by RNAPII, EWS, and YB-1. (a) In normal condition EWS associates with the preinitiation complex TFIID, with the RNAPII and with its target transcripts and associated genomic regions; once the transcription has started, EWS jumps on the nascent RNA promoting specific AS events. (b) Upon CPT treatment EWS dissociates from YB-1 and from the spliceosome [64]. Similarly, upon low intensity UV light irradiation EWS dissociates from active transcription sites and relocalizes to nucleoli [51]. As a result, EWS regulated AS events cannot occur [51, 64].

Changes in RNAPII elongation rates modulate the timing at which competing splice sites become available, and it is another coupling mechanism that could possibly be used by EWS. The interaction of EWS with some of the earliest factors involved in 5′ and 3′ splice site recognition (i.e., U1 snRNP and BBP/SF1 [58, 60]) opens the possibility that EWS establishes initial links between splice sites across introns or exons that contribute to splice site selection and exon definition (Figure 2(a)). 

More recently, two studies have directly linked EWS function to AS of specific genes in cancer cells. EWS was shown to regulate AS of the p53 repressor (Mouse Double Minute 2, Human Homolog) *MDM2* [64] and several other genes involved in the DNA damage response (DDR) [51]. Camptothecin (CPT) treatment inhibits the interaction between EWS (associated with RNAPII) and YB-1 (a spliceosome-associated factor), thus resulting in general exon skipping both in *MDM2* and in other genes. These AS events parallel the events induced either by EWS or YB-1 knockdown (Figure 2(b), left scheme) [64], suggesting that CPT inhibits the splicing activity of these two RNA binding proteins (RBPs). Similarly, UV irradiation causes EWS dissociation from genomic and transcription sites, concomitant with increased translocation of the protein in nucleoli [51]. This mechanism contributes, at least in part, to the AS changes detected upon EWS knockdown or UV irradiation (Figure 2(b)) [51]. These reports document that EWS binds directly to the alternatively spliced regions of its target genes, suggesting a direct role for EWS on the regulation of these events (Figures 2(a) and 2(b)). The reported correlation between CLIP (cross-linked and immunoprecipitation) and ChIP (chromatin immunoprecipitation) signals further suggests a direct association of EWS with DNA, as also confirmed by EMSA experiments [51]. EWS binding was also observed in promoter regions, although not specifically for genes displaying AS regulated by EWS [51]. Moreover, the partial decrease in ChIP signals upon RNase treatment suggests that EWS associates with its target transcripts cotranscriptionally (Figure 2(a)) [51]. 

Collectively these observations confirm the dual function for EWS in transcription and RNA processing and suggest a potential role for this protein in the coupling between the two processes. However, the specific mechanism by which EWS exerts splicing regulation has not been unraveled yet. 

## 8. EWS and Noncoding RNAs

EWS protein, as well as the other members of TET family, has been identified as part of a multiprotein complex associated with DROSHA [70], the RNase III endonuclease assigned to the cleavage of the primary microRNA (pri-miRNA) [71]. DROSHA is a component of two multiprotein complexes. The larger complex contains multiple classes of RBPs including RNA helicases, proteins that bind double-stranded RNA, several hnRNPs, and, as mentioned above, the TET family of RBPs. The smaller complex, composed by DROSHA and the double-stranded-RNA-binding protein DGCR8, forms the microprocessor complex, necessary and sufficient for the genesis of miRNAs from the pri-miRNA transcripts [70]. Since DROSHA is required for miRNA biogenesis and its activity can be modulated by regulatory proteins, TET proteins might play a role in miRNA expression by modulating the activity of this processing enzyme. Interestingly, some evidence has recently linked EWS to microRNA processing. Depletion of EWS in osteosarcoma U2OS cells results in the accumulation of precursor let-7g and downregulation of mature let-7g [72]. These findings lead to the hypothesis that EWS might play a role in miRNA biogenesis and maturation. Nevertheless, wide spectrum analyses of the impact of EWS on miRNA expression are still lacking; thus its contribution to this process is still largely unknown.

In addition, TET proteins have been recently involved in the regulation of other classes of noncoding RNAs. It has been shown that the interaction of FUS/TLS with CBP/p300 is induced by ncRNAs transcribed from the promoter regions of *CCND1 *gene. These ncRNAs bind to FUS/TLS and inhibit CBP/p300 histone acetyltransferase (HAT) activity [73]. Similarly, EWS and TAF15 were found to bind to CBP and to exert inhibitory effects on CBP/p300 HAT activities [73]. The binding of EWS and FUS/TLS to G-quadruplex structures is the key of this regulation. Upon binding to GGUG RNA oligonucleotides in ncRNAs, the inhibitory activity of FUS/TLS against the HAT activity of CBP/p300 is enhanced. Biochemical experiments showed that the carboxy terminus of FUS/TLS binds to GGUG RNA, whereas the N-terminus interacts with CBP. The interaction of FUS/TLS with CBP/p300 is stimulated when FUS/TLS is bound to G-quadruplex, probably due to a conformational change, and this in turn results in inhibition of CBP/p300 HAT activity [73]. 

Similar to FUS/TLS, EWS can bind to TERRA [48, 53], a large noncoding RNA component of telomeric heterochromatin, which inhibits the telomerase activity [74]. Also in this case, the interaction occurs through the G-quadruplex in a structure-specific manner [53]. Thus, although not investigated in detail yet, binding of EWS to ncRNAs appears to contribute to its function in gene expression regulation.

## 9. EWS and Genotoxic Stress

Genomic integrity is critical to cell survival and is controlled by the DDR network, an elaborate signal transduction system that senses DNA damage and recruits appropriate repair factors [75].

As mentioned above, *Ews* deficiency in mice leads to developmental defects in meiosis and pre-B cell formation [23]. Interestingly, defects in lymphocyte development, meiosis, and cellular senescence (or aging) are features also observed in mice deficient in *Atm* and *c-abl *[76, 77], two genes that are critical for the DDR pathway. These observations implicate a possible role of EWS in the DNA recombination and/or repair process. Homologous recombination during meiosis is a physiological process that requires DNA double-strand breaks and repair. It is essential for the proper segregation of chromosomes and it is regulated by most of the genes that are also involved in DNA repair after genotoxic stress, as revealed by the gametogenesis defects observed in several knockout mouse models of genes involved in the DDR [78]. It was reported that in the absence of EWS the meiotic crossovers are conspicuously reduced or even absent in some of the bivalents. This finding demonstrated that EWS is critically important for the homologous recombination during meiosis and suggested its possible involvement in the DDR pathway [23]. Indeed, consistent with the hypothesis, *Ews*-null mice and mouse embryonic fibroblasts (MEFs) showed increased sensitivity to ionizing radiation [23]. 

A physiological function for EWS in response to genomic alterations is consistent with two recent genetic screens that identified *EWSR1* as a gene required for resistance to ionizing radiations (IR) [79], which release intermediary ions and free radicals that cause DNA lesions, and to CPT, a topoisomerase I inhibitor which forms a tight complex with topoisomerase I-DNA adducts and prevents DNA religation, thus leading to formation of single-strand breaks (SSBs) [80]. Further evidence in support of its involvement in the DDR is the reported phosphorylation of EWS on threonine 79 upon DNA damage [81]. This posttranslational modification occurs in response to both mitogens and DNA alkylating agents and is catalyzed by mitogen-activated protein kinases (MAPKs); however, while the phosphorylation upon mitogen signals is operated by the ERK1/2 (extracellular signal-regulated kinases 1 and 2), DNA damage induced phosphorylation of EWS is catalyzed by the JNK1/2 (extracellular signal-regulated kinases 1 and 2) [81]. Interestingly, phosphorylation on threonine 79 occurs also in the oncogenic EWS-FLI1 protein upon treatment with DNA alkylating agents and is operated by p38*α*/p38*β* MAPK [81]; nevertheless, the functional relevance of this phosphorylation has not been unraveled yet. 

Moreover, EWS protein has also been described to interact with BARD1, a putative tumor suppressor component of the BRCA1/BARD1 complex that performs essential DNA repair and recombination functions [82]. Altogether, the observations reported above suggest that, in addition to its involvement in endogenous processes of homologous recombination, EWS plays also a role in DDR triggered by genotoxic stresses.

In addition to the well-described activation of a signal transduction pathway that regulates cell cycle progression and apoptosis, low-intensity UV light induces also extensive changes in AS driven, at least in part, by hyperphosphorylation of RNAPII carboxyterminal domain (CTD) and reduced transcription elongation [83]. Moreover, we have recently documented that UV light influences AS of a subset of genes involved in the DDR, such as *BRCA1, CHEK2,* and *ABL1,* through regulation of the activity of EWS [51]. DNA repair and checkpoint responses are critically important to ensure the integrity of the genome. Checkpoint pathways are composed of sensors that detect damaged or unreplicated DNA, transducers that convey the signal, and effector proteins that act on the ultimate targets of the checkpoint. CHK2 (the protein product of the *CHEK2* gene) is an effector protein conserved in eukaryotes and phosphorylated by the sensor kinases ATM/ATR in response to DNA damage; upon phosphorylation by ATM/ATR, CHK2 phosphorylates and thus modifies the function of key targets of the checkpoint response [84]. Interestingly, upon EWS knockdown an mRNA isoform of *CHEK2* is generated that lacks the exon 2 containing the translation initiation codon, thus leading to a decrease of the levels of CHK2 protein [81]. These findings suggest that proper levels and activity of EWS are important to maintain CHK2 expression and, given the key role of this kinase in DNA damage response, for the initial cellular response to genotoxic stress. EWS knockdown could therefore lead to defective CHK2 response and explain the higher sensitivity to UV irradiation.

Interestingly, UV light induces dissociation of EWS from sites of active transcription, in particular from alternatively spliced regions regulated by this protein, and its translocation to the nucleoli [51]. Similar translocation to the nucleolar compartment has been described also for FUS/TLS upon inhibition of transcription [85]. As mentioned above, EWS is also involved in other genotoxic stress-mediated changes in AS regulation. It was shown that treatment with CPT leads to substantial exon skipping and that a subset of the triggered AS events could be recapitulated by knockdown of EWS or of its interacting protein YB-1 [64].

Collectively, these observations highlight the role of EWS in the DDR and suggest that this protein acts, at least in part, through the regulation of AS of genes involved in the activation of the DNA damage checkpoint and for the repair of DNA lesions. Nevertheless, since EWS binds DNA and it is found associated with the chromatin [23, 51], it is also possible that it plays a direct role in DNA recombination and repair. 

## 10. EWS and Neuromuscular Disorders

Regulation of RNA metabolism in the nervous system plays a crucial role, as highlighted by the development of devastating neurological diseases, including spinal muscular atrophy and certain trinucleotide repeat expansions, upon mutation or misregulation of key RBPs. Mutations in FUS/TLS, as well as in the RBP TAR DNA-binding protein 43 (TDP-43), were found to be causative of familial cases of amyotrophic lateral sclerosis (ALS) [15, 16, 86], further implicating altered RNA metabolism as a central feature of these diseases. FUS/TLS protein was also reported to be the major component of poly-Q aggregates in cellular models of spinal cerebellar ataxia and in intracellular inclusions in neurons of patients with Huntington's disease [87]. Indeed, TDP-43 and FUS/TLS [88], as well as TAF15 and EWS [86], are recruited in cytoplasmic aggregates in frontotemporal lobar degeneration (FTLD). Thus, aggregation of RBPs is emerging as a crucial event in clinicopathological conditions of neurodegenerative diseases. Since all these RBPs are directly involved in RNA processing and utilization, these observations highlight RNA dysmetabolism as potential key step underlying neurodegenerative diseases. Intriguingly, many of these RBPs that aggregate in pathological inclusions are expressed broadly, some even ubiquitously, but they seem to only aggregate in certain neurons and not others, remarking cell type specificity [89]. 

Most disease-causing mutations in TDP-43 and some disease-causing mutations in FUS/TLS are found within the glycine-rich domain, which is rich in uncharged, polar amino acids, similar to that observed in the nucleation domains found in many yeast prions [90]. This has led to speculation that a prion-like aggregation property of some RBPs may contribute to onset and/or progression of ALS and related neurodegenerative diseases [91]. The majority of yeast prion proteins contain a distinctive prion domain that is enriched in uncharged polar amino acids (particularly asparagine, glutamine, and tyrosine) and glycine [92, 93]. An algorithm designed to detect prion domains has identified 19 domains that can confer prion behavior [90]. Scouring the human genome with this algorithm enriches a select group of RBPs harboring a canonical RRM and a putative prion domain. Indeed, of the 210 human RRM-bearing proteins, 29 have a putative prion domain and these prion RBPs are emerging, one by one, in the pathology of devastating neurodegenerative disorders [91, 94].

Similar to *TDP-43*, *FUS*/TLS, and *TAF15*, expression of *EWSR1* cDNA in yeast resulted in cytoplasmic aggregation and toxicity in a functional screen [94]. Furthermore, like *TDP-43*, *FUS/TLS,* and *TAF15*, *EWSR1* also harbors a bioinformatically predicted prion-like domain. Since most of the pathogenic mutations in FUS/TLS are located in the C-terminal domain of the protein, the last four exons of the *EWSR1* gene (exons 15–18) were sequenced in 817 individuals diagnosed with ALS and in 1082 healthy population control individuals. This approach identified two patient-specific missense variants in exon 16 (1532G>C, G511A, corresponding to the first RGG domain) and in exon 17 (1655C>T, P552L, corresponding to the second RGG domain) of *EWSR1* in two unrelated ALS patients with sporadic disease. Interestingly, these variants alter EWS localization in motor neurons and exhibit enhanced aggregation propensity [95]. Nevertheless, the role played by EWS in ALS and in other neurological diseases still remains unveiled, and further studies are needed to identify the molecular targets affected in these pathological conditions. Notably, whether EWS RNA binding affinity and pre-mRNA regulation are affected in the ALS-related mutants is also still unknown.

## 11. EWS and Cancer

Much knowledge about the function of EWS is derived from studies of oncogenic fusion proteins involved in several malignancies and arising from in-frame chromosomal fusions with multiple partners. However, besides the oncogenic translocations, an independent role for EWS in cancer has not been unraveled yet. 

Gene expression in cancer cells is deregulated at both the transcription and splicing levels, and there are many examples of oncogenic and cancer-associated splice variants [96]. As described above, the regulation of specific splicing events and transcriptional events by EWS might be involved in the altered transcriptome of cancer cells [51, 64, 66, 69]. 

Cancer progression is thought to be dependent on the accumulation of mutations that change the transcriptional profile of the cell to support its escape from the tight regulation of cell cycle progression. Improper responses to different types of DNA lesions may lead to accumulation of mutations in the genome, which then accelerate the progression of the disease. Thus, given the reported role of EWS in genome stability, it is possible that this RBP is also essential to preserve the genome integrity and protect cells from neoplastic transformation. 

Accordingly, EWS plays a critical role in the DDR [51, 64], and *EWSR1* gene has been shown to be essential for a proper response to genotoxic agents [23, 79, 80]. In particular, EWS regulates AS of genes playing a key role in oncogenesis [51] like *CHEK2* (described above), *BRCA1* (breast cancer 1 gene), and *ABL1. BRCA1* is a tumor suppressor and germline mutations in *BRCA1* gene predispose individuals to breast and ovarian cancers [97]. Interestingly, *BRCA1* gene generates several splice variants that play an important role in tumor development [97]. Mutations have been described in *BRCA1* gene that result in skipping of more than one exons leading either to shorter transcripts or to frameshift and premature stop codons, thus rendering mis-spliced mRNAs subject to non-mediated decay (NMD). Thus, these mutations yield either a truncated BRCA1 protein or loss of a *BRCA1* transcript. As a result the functionality of the resulting BRCA1 protein is severely compromised. Remarkably, EWS knockdown in HeLa cells results in aberrant isoforms of *BRCA1* gene lacking the exons from 12 to 17 [51]. 

*EWSR1* knockdown in HeLa cells resulted also in a significantly high incidence of abnormal spindles and mislocalization of Aurora B kinase, the enzymatic core of the chromosomal passenger complex (CPC), which orchestrates in space and time chromosome alignment, histone modification, and cytokinesis [98]. Proper localization of the CPC complex is key to the precise control of chromosome segregation over mitosis, and dysregulation of Aurora kinase activity has been linked to tumorigenesis [99]. Indeed, the absence of Aurora B results in increased numbers of aneuploid cells, genetic instability, and oncogenic transformation [99].

On the other hand, the oncogenic potential of EWS in the chimeric protein is driven by its N-terminal transactivation domain, which is partially inhibited by the RGG domains in the C-terminal part of the full-length protein. So the C-terminal part of EWS protein, missing in the fusion oncogenes, could be strategically relevant in protecting cells from cancer either by playing a key role in the regulation of AS of DDR genes or by inhibiting the DNA activation domain or through both mechanisms.

Interestingly, it has been shown that in the nucleus EWS interacts with STRAP (serine-threonine kinase receptor-associated protein), a WD40 domain-containing protein. EWS-STRAP interaction is strategically important for STRAP-induced inhibition of EWS transactivation function. Strikingly, STRAP is upregulated in colon and lung carcinomas [100] and STRAP upregulation in colon tumors correlates with EWS expression [101]. Furthermore, STRAP blocks EWS-induced p300-mediated expression of c-fos by interfering with EWS-p300 interaction, inhibiting in this way EWS-mediated transactivation [101]. Thus, the cooperation between EWS and STRAP could be involved in tumor progression and interfering with EWS-mediated transactivation could be a mechanism of regulation of its transcriptional properties in human cancers. 

## 12. Conclusive Remarks

EWS protein is involved in the regulation of a wide range of cellular processes to ensure genome integrity and proper pursuance of cellular functions. Early studies suggested a role for EWS in transcription and splicing, possibly coupling these two processes. Since then, many studies have unraveled these functions and unveiled new roles for EWS protein in other aspects of the regulation of gene expression and RNA metabolism. Importantly, some RNA targets and new interacting proteins have now been identified [102]. Moreover, new insights have been provided on the regulation of the amino terminus of EWS, which is involved in oncogenic fusion proteins and responsible for the inappropriate transcriptional activation of target genes. 

More recently, novel potential functions have been suggested for EWS protein in cancer and in neuromuscular disorders, disclosing novel roles for the protein. Nevertheless, there are still many important unsolved questions concerning the molecular and cellular biology of EWS and further knowledge is needed to understand if EWS represents a suitable target for the development of new approaches to cancer therapy.
